# Risk factors associated with the detection of pulmonary emphysema in older asymptomatic respiratory subjects

**DOI:** 10.1186/s12890-020-01204-9

**Published:** 2020-06-09

**Authors:** Ivette Buendia-Roldan, Alexia Palma-Lopez, Danaireth Chan-Padilla, Iliana Herrera, Mariel Maldonado, Rosario Fernández, David Martínez-Briseño, Mayra Mejia, Moises Selman

**Affiliations:** 1 Instituto Nacional de Enfermedades Respiratorias “Ismael Cosío Villegas”, Tlalpan 4502, CP 14080 Ciudad de Mexico, Mexico; 2 Universidad Autónoma de Sinaloa, Facultad de Medicina, Culiacán, Mexico

**Keywords:** Aging, Pulmonary emphysema, COPD, Risk factors, Klotho, Telomere length

## Abstract

**Background:**

Several lung structural and functional abnormalities may occur associated with aging, including emphysema. In this study, we evaluated the frequency and risk factors associated with emphysema in respiratory asymptomatic individuals enrolled in our Lung Aging Program. From a cohort of 687 subjects, we found by high-resolution computed tomography (HRCT) 29 individuals (4%) with emphysematous changes that were compared with 87 controls (3:1) randomly selected from the same cohort.

**Methods:**

This was a transversal, observational, case-control study where we examined demographics and functional characteristics, as well as telomere length and serum Klotho concentration, two conditions that have been associated with aging and some aging-associated diseases including emphysema.

**Results:**

Individuals with subclinical pulmonary emphysema were older (72 ± 9 versus 67 ± 6 years), and primarily smoker males with low body mass index. Despite that they were asymptomatic, two of them exhibited a decrease of forced expiratory volume in 1 s (FEV_1_), with a lower FEV_1_/FVC suggesting airway obstruction. Cigarette smoking (OR = 5.43, CI95% 1.8–16.7), family history of lung disease (OR = 4.32, CI95% 1.0–19.0) and lower body mass index (OR 7.22, CI95% 1.2–3.5) were risk factors for the development of lung emphysematous changes. No association was found with telomere length and Klotho serum concentration.

**Conclusion:**

Our findings reveal that a small but important percentage of older people without respiratory symptoms, present pulmonary emphysema and indicate that smoking exposure and genetic background may contribute to etiological factors.

## Introduction

Aging is a normal biological process associated with multiple anatomic and functional abnormalities and morbidities. The physiological effects of aging in the lungs include, among others, a progressive decrease in forced vital capacity with an increase of pulmonary vascular resistance [1]. The lungs of older people may also show interstitial lung abnormalities, decreased elastic recoil and decreased diameter of the small airways with the premature close of the peripheral airways [2].

Some individuals develop changes in the lung structure with an increase in the size of the alveolar spaces (over-distension) without inflammation or alveolar wall destruction, so-called “senile emphysema” a term that has been discarded [3]. By contrast, some individuals mostly smokers may develop “real” emphysema characterized by alterations of the extracellular matrix, destruction of the alveolar walls and loss of the lung architecture [3, 4]. These changes are usually, but not always associated with airways alterations constituting the chronic obstructive pulmonary disease (COPD).

The presence of emphysema as the only alteration is uncommon and has been mainly associated with alpha-1-antitrypsin deficiency [5]. However, the frequency and pathogenic mechanisms of emphysema in asymptomatic older individuals without a known genetic etiology remains uncertain.

In the last years, we have been working on a “Lung Aging Program” involving respiratory asymptomatic individuals > 60 years, both smokers and non-smokers, who have lived in Mexico City at least during the last 10 years (altitude ~2500mts over sea level).

In this cohort, we found by high-resolution computed tomography (HRCT) 4% of individuals with emphysematous changes affecting > 5% of the lung parenchyma. In this context, the aim of our study was to evaluate demographics and functional characteristics, as well as telomere length and serum Klotho concentration in individuals with subclinical pulmonary emphysema compared with controls randomly selected from the same cohort, to identify if they could be risk factors to develop this disease. The length of telomeres and levels of Klotho was selected because telomere dysfunction and a marked decrease of circulating Klotho have been associated with COPD [6, 7] but their association in subclinical pulmonary emphysema was not clear.

## Methods

### Study population

Our Aging Lung Program voluntarily enrolls respiratory asymptomatic individuals over 60 years through an open invitation, that has been living in Mexico City for at least 10 years. The cohort includes current, former and non-smoker subjects, chronic systemic diseases (arterial hypertension, diabetes, etc. with medical control). Individuals with body mass index (BMI, kg/m^2^) lower than 18.5 and obese (BMI > 30) are excluded. This program is being developed in the Instituto Nacional de Enfermedades Respiratorias since march 2015.

Several questionnaires are applied to evaluate demographic, and health and respiratory status, including PLATINO, a composite instrument that has sections of ATS/DLD, ECRHS II, Lung Health Study, and SF-12 [8],

All individuals perform pulmonary function tests including forced vital capacity (FVC), forced expiratory volume in 1 s (FEV_1_), diffusing capacity of the lung for carbon monoxide (DL_CO_%) corrected for altitude, and six-minutes’ walk test, changes in oxygen saturation and walked distance are measured.

On the same day, individuals underwent high resolution computed tomography (HRCT) helicoidal scanning (Somatom, definition AS 128 detectors double-energy, Siemens), in supine and prone position and the interpretation of images is performed by two pulmonologists and one radiologist with at least 10 years of experience.

**Fig. 1:**
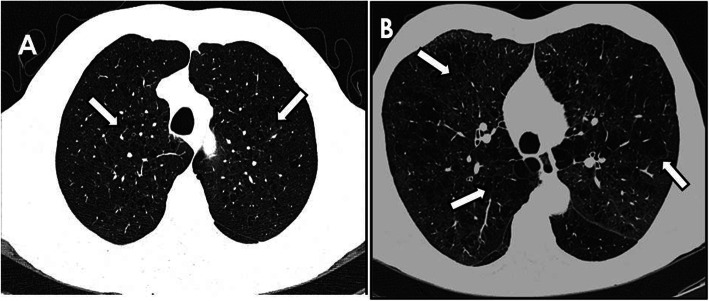
**a** and **b** show high resolution computed tomography of two different respiratory asymptomatic individuals with different severity of emphysematous lesions (arrows)

From our current cohort of 687 respiratory asymptomatic individuals, 29 showed HRCT image features of pulmonary emphysema (Fig. 1). These individuals were compared with 87 controls from the same cohort (3:1) randomly selected. A signed informed consent letter was obtained from all participants of the Program, and the study was approved by the Research and Ethics Committees of National Institute of Respiratory Diseases, Mexico (number of approved C39–14).

### Analysis of telomere length by quantitative real-time PCR

Relative telomere length was measured by quantitative polymerase chain reaction (qPCR) as previously described [9]. Genomic DNA was extracted from blood samples and reactions were performed with the following reagents: Power SYBR® Green PCR Master Mix (Life Technologies, UK), RNase free water (SIGMA, UK), primer single gene (S) forward (36B4d F-300 nM) (CCCATTCTATCATCAACGGGTACAA) and single-copy gene (S) reverse (36B4u R-300 nM) (CAGCAAGTGGGAAGGTGTAATCC), primer Tel (T) Forward (900 nM) (CGGTTTGTTTGGGTTTGGGTTTGGGTTTGGGTTTGGGTT), Tel (T) Reverse (900 nM) (GGCTTGCCTTACCCTTACCCTTACCCTTACCCTTACCC). The cycling profile was: 95 °C for 10 min; 95 °C for 15 s, 58 °C for 1 min, 72 °C for 30 s × 40 cycles; 95 °C for 15 s, 55 °C for 15 s and 95 °C for 15 s. Outlier values were excluded. The relative value for telomere length telomere repeats copies number (T) to a single copy gene (S) (T/S) ratio was determined by comparison with control calibration curves and was graphed as natural logarithm (LN) versus age.

### Serum levels of soluble α-klotho

α-Klotho was evaluated in serum by enzyme-linked immunosorbent assay (ELISA) using a commercial kit (IBL América, Cat 279,988) according to the manufacturer’s protocol.

### Statistical analysis

Results were expressed as mean ± standard deviation. A comparison between groups was performed by Fisher’s exact for categorical values and U-Mann Whitney for continuous variables; significance was defined as *P* < 0.05. Multivariable logistic regression was used to assess factors associated with emphysema using the program Stata for Windows, version 12.

To obtain the correlation between relative telomere length and age, linear regression was performed using integrated data analysis in GraphPad Prism v6 (GraphPad Software Inc., CA, USA). The natural log-transformed relative T/S ratio was normally distributed.

## Results

### Demographic characteristics

Individuals with pulmonary emphysema were older male and primarily former cigarette smokers compared with the non-emphysema control group (Table 1). They also showed a lower body mass index compared with controls. Interestingly, subjects with emphysema had more often a history of relatives with some chronic lung disease (Table 1). The type of emphysema was predominantly centrilobular (79%). Four percent showed pan-lobular emphysema and 17% was mixed.

**Table 1:** Demography factors and co-morbidities

Variable	Emphysema n = 29	Control n = 87	p
Gender, (male: female)	22:7	23:64	< 0.0001
Chronological age, years (SD)	72 ± 9	67 ± 6	0.004
Body mass index (SD)	24 ± 3	27 ± 4	0.0007
Cigarette smoking, former (%)	23 (79)	36 (41)	< 0.0001
Occupational exposure, (%)	15 (52)	35 (40)	0.279
Family history with lung disease, (%) ^a^	5 (17)	4 (5)	0.02

^a^Reported lung disease = Chronic obstructive pulmonary disease, chronic bronchitis, fibrosis, emphysema. SD = standard deviation

### Pulmonary function tests

Two of the individuals with emphysema exhibited a decrease of FEV_1_ (45 and 46% percent predicted), with a lower FEV_1_/FVC indicating airway obstruction. These two individuals also showed a lower DL_CO_ (66 and 64% percent predicted), and one of them displayed oxygen desaturation after exercise. The rest of the subjects have normal spirometry without differences with the control group (Table 2). However, as a group, even removing the two subjects with a significant decrease of DL_CO_, individuals with emphysema showed a lower DL_CO_ but without differences in oxygen saturation and walked distance after exercise (Table 2).

**Table 2:** Lung function test results

	Emphysema n = 29	Control n = 87	p
FVC, %predicted, (± SD)	93 ± 15	96 ± 16	0.06
FEV1, %predicted, (± SD)^a^	95 ± 16	100 ± 17	0.2
FEV1/FVC %, (±SD)	101 ± 11	104 ± 8	0.1
DL_CO,_ % predicted, (±SD)^a^	104 ± 20	115 ± 20	0.01
SpO2 at rest, % (±SD)	94 ± 2	94 ± 2	0.7
Meters 6-MWT, (±SD)	91 ± 6	92 ± 4	0.3

*FVC* Forced vital capacity, *FEV1* Forced expiratory volume in one second.

*DL*_*CO*_ Diffusing capacity of the lung for carbon monoxide. 6-MWT = Six-minutes walking test. *SD* Standard deviation.

^a^These data do not include two patients with airway obstruction (see Results)

### Leukocyte telomere length

**Fig. 2:**
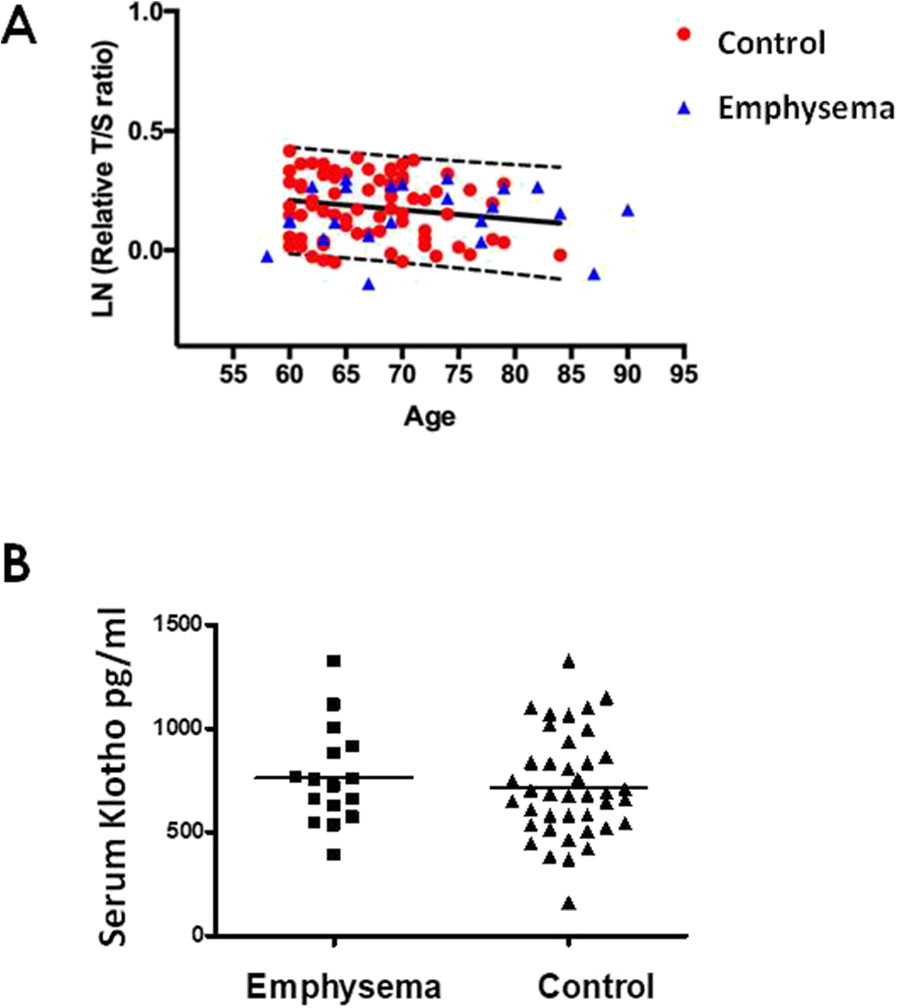
**a** Linear regression analysis of telomere length and age in individuals with emphysema and controls. Telomere length measured by qPCR from control samples (red circles, n = 83) and subject with emphysema (blue triangles, n = 27) was plotted relative to age. The area between the lines delineates the 10th to 90th percentile predicted bands. LN: natural logarithm of T/S ratio. **b** Serum levels of Klotho in subjects with pulmonary emphysema and controls

Since abnormal shortening of telomeres has been associated with COPD [6] we wonder whether telomere length was also associated with the presence of subclinical pulmonary emphysema. However, as shown in Fig. 2A, no significant difference between the control and emphysema groups was detected.

### Serum concentration of klotho

Low levels of soluble Klotho have been also associated with COPD [10]. In this context, we evaluated serum Klotho concentrations by ELISA. As shown in Fig. 2B**,** no significant differences were found between individuals with subclinical pulmonary emphysema (762.6 + 238 pg/ml) and controls (695.02 + 287 pg/ml; *p* = 0.15).

### Risk factors

**Fig. 3:**
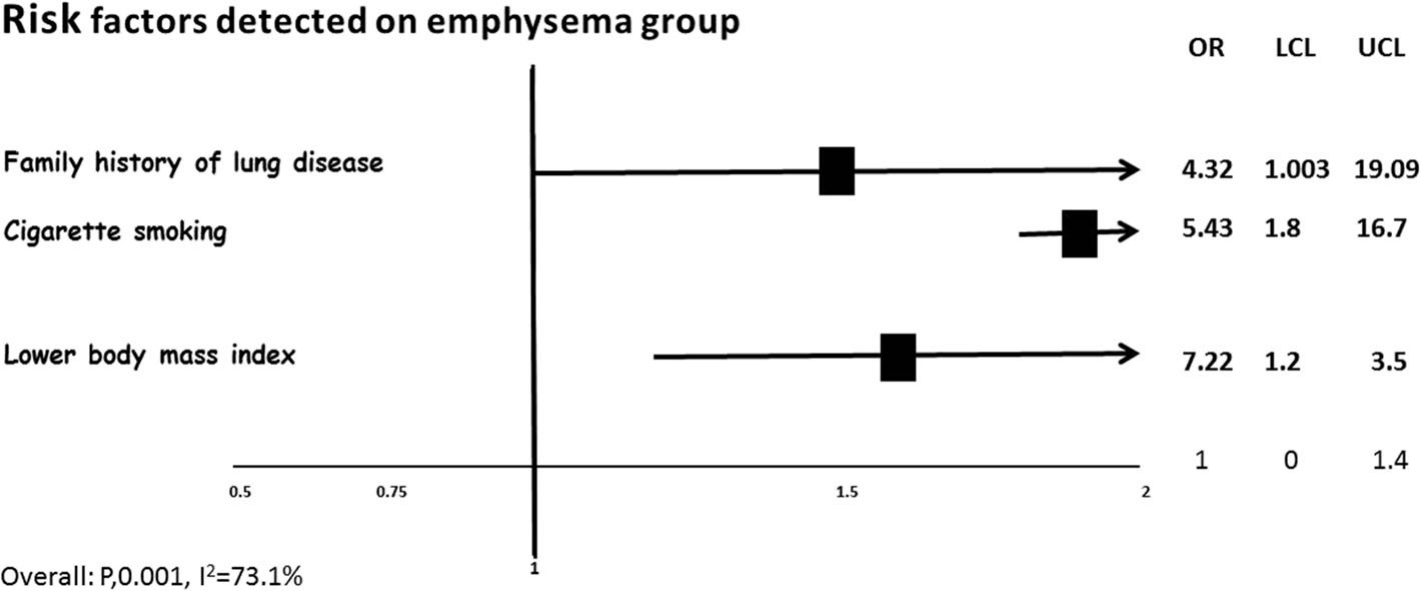
Risk factors for aging-associated with pulmonary emphysema

Three risk factors were significantly associated with the presence of emphysema, family history of lung disease [OR 4.32 (CI95%1.003–19.09)], cigarette smoking [OR 5.43 (CI95% 1.8–16.7)], and lower body mass index [OR 7.22 (CI95% 1.2–3.5)] (Fig. 3). Family lung disorders included airways and parenchymal diseases such as chronic obstructive pulmonary disease, chronic bronchitis, emphysema, and pulmonary fibrosis. A non-significant tendency was found with the history of environmental and occupational exposure [OR 1.59 (CI95% 0.63–4.03)].

## Discussion

Pulmonary emphysema represents a form of destruction of the lung architecture characterized by an abnormal and permanent enlargement of the air space distal to the terminal bronchioles, with the destruction of the alveolar walls, and without obvious fibrosis [11]. Emphysema, usually as part of COPD, represents a slowly progressive and irreversible lung disorder, resulting in respiratory insufficiency and reduction in life expectancy and life quality. Pulmonary emphysema may occur associated with gene mutations such as alpha1-antitrypsin and telomerase components [12, 13], but the sporadic form associated with COPD, is primarily related with the exposure to cigarette smoke and other respiratory environmental or occupational exposures such as gases, biomass smoke, fumes and dust [14].

Unfortunately, the onset and progression of emphysema, COPD and other lung diseases associated with aging are insidious and are often misdiagnosed leading to irreversible damage before diagnosis. Thus, many patients are identified as having ‘smoker’s cough’, asthma or bronchial infection and are diagnosed too late.

Importantly, HRCT represents a consistent diagnostic tool even for subtle modifications in secondary pulmonary lobules, then allowing early diagnosis. Likewise, pulmonary function tests may help the early detection of these age-associated disorders. Actually, it has been suggested to perform community-based spirometry to find patients with early disease, mainly in smoker individuals over 40 years old who present with lower respiratory tract symptoms [15].

However, HRCT screening is more sensitive than lung function tests for emphysema detection, because it may show structural changes even with no airway obstruction [16]. Our study corroborates this notion because we were able to detect emphysematous changes in the lungs of individuals without airway obstruction.

In our study, we found that from almost 700 individuals evaluated so far, around 4% of them, without respiratory symptoms, show pulmonary emphysema by HRCT. Only two of them show a significant decrease in FEV_1_ and FEV_1_/FVC ratio, indicating airway obstruction. Interestingly, even excluding them, individuals with emphysema exhibited decreased DL_CO_ suggesting an early alteration in gas exchange (Table 2).

Detecting subclinical emphysema that is lung alterations in the early stages, (e.g., respiratory asymptomatic individuals such as in our study) can help to provide timely preventive interventions and treatment, avoiding long term complications and improving the quality of life of people with chronic respiratory disorders. For example, it has been found that the detection of mild emphysema with normal functional pulmonary tests in young smokers led to negative impacts on their quality of life [15].

We were also interested in detect risk factors and putative biomarkers. As expected, the frequency of pulmonary emphysema was higher in cigarette smokers, which has been clearly identified as its major risk factor. However, this disorder was also observed in never smokers indicating that other risk factors are involved, and our results show that family history of lung disorders also influences the risk to develop emphysema suggesting some inherited susceptibility. This finding agrees with studies in large cohorts which indicate that family history of COPD is a strong risk factor for the development of the same disease, independent of personal lifetime smoking, or childhood environmental tobacco smoke exposure [17, 18].

Individuals with emphysema were chronological older compared with controls, but interestingly, the highest phenotypic age relative to the chronological age also seemed to be associated with emphysema. This is an important observation since it has been previously demonstrated that Phenotypic Age, a novel clinically-based measure of aging, was predictive of mortality among both healthy and unhealthy populations even after adjusting for chronological age [19, 20].

We also investigated whether the circulating concentrations of Klotho, an anti-aging molecule, or the leukocyte telomere length are associated with the risk for pulmonary emphysema, but no differences with the control group were detected. These findings suggest that alterations in these two molecules are noticeable in more advanced disease.

This study has several limitations. First, the sample size was small and the number of molecular evaluations restricted. Second, the studied population resides in Mexico City at a higher latitude and pollution than many other cities and in this context, their effects on our findings remain uncertain. Also, telomere length was measured by qPCR instead of quantitative fluorescence in situ hybridization (qFISH).

However, our findings support the implementation of screening studies in subjects over 60 years with associated risk factors, even when they do not have respiratory symptoms. Since emphysema has a long evolution before produce symptoms, it would be clinically relevant to detect the disease when lung destruction is limited and smoking cessation and other programs may prevent progressive functional impairment.

## Conclusions

This study reveals that a small but significant percentage of older, respiratory asymptomatic individuals present emphysematous lesions that may be diagnosed earlier mainly if they have a history of smoking and a family history of lung diseases.

## Data Availability

The datasets used and/or analysed during the current study are available from the corresponding author on reasonable request.

## Abbreviations

ATS: American thoracic society
BMI: Body mass indez
CI: Confidence interval
COPD: Chronic obstructive pulmonary disease
DL_CO_: Diffusing capacity of the lung for carbon monoxide
DLD: Division of lung disease
DNA: Deoxyribonucleic Acid
ECRHS: European community respiratory health survey
ELISA: Enzyme-linked immunosorbent assay
FEV_1_: Forced expiratory volume in 1 s
FVC: Forced vital capacity
HRCT: High-resolution computed tomography
LN: Natural logarithm
OR: Odds Ratio
PLATINO: Latin-American research project in pulmonary obstruction
qFISH: Quantitative fluorescence in situ hybridization
qPCR: Quantitative polymerase chain reaction
SD: Standard deviation
SF: Short form
T/S: Telomere repeats copies number to a Single copy gene
